# The Effects of Endurance Training and High-Intensity Resistance Training on Pulse Wave Velocity and QT Dispersion

**DOI:** 10.3390/healthcare13020161

**Published:** 2025-01-15

**Authors:** Selma İşler, Metin Çoksevim, Tülin Akman, Şaban Ünver, Burçin Öner, Ayhan Bilgici

**Affiliations:** 1 Faculty of Medicine, Physical Medicine and Rehabilitation, Ondokuz Mayis University, 55270 Samsun, Turkey; abilgici@omu.edu.tr; 2 Faculty of Medicine, Cardiology, Ondokuz Mayis University, 55270 Samsun, Turkey; metin.coksevim@omu.edu.tr; 3 Department of Coaching Education, Faculty of Sports Science, Ondokuz Mayis University, 55270 Samsun, Turkey; takman@omu.edu.tr (T.A.); saban.unver@omu.edu.tr (Ş.Ü.); 4 Bozüyük Vocational School, Bilecik Şeyh Edebali University, 11300 Bozüyük, Turkey; burcin.oner@bilecik.edu.tr

**Keywords:** arterial stiffness, pulse wave velocity, QT dispersion, electrocardiography

## Abstract

**Objectives:** This study aimed to examine the effects of endurance and high-intensity resistance training on arterial stiffness and ventricular repolarization in elite athletes. **Methods:** A total of 50 male athletes from different sports disciplines (volleyball, football, judo, and wrestling) and a sedentary group of 30 males participated in this study. Data collected from all participants included age, height, body weight, cardiovascular hemodynamic parameters, arterial stiffness parameters, and ECG measurements. **Results:** There was no significant age difference between the athlete group (20.42 ± 1.903 years) and the control group (20.97 ± 1.771 years) (*p* > 0.05). However, body mass index (BMI) values in the control group (24.83 ± 2.22 kg/m^2^) were significantly different from those in the athlete group (22.39 ± 2.663 kg/m^2^) (*p* < 0.05). Significant differences were found between the athlete and control groups in QT dispersion, systolic blood pressure, pulse pressure, and central pulse pressure values (*p* < 0.05), while similar results were obtained for arterial stiffness parameters (*p* > 0.05). **Conclusions:** The lack of a difference in pulse wave velocity and augmentation index (AIx) values between the athletes and the control group suggests that athletes do not bear additional risks regarding arterial stiffness. However, increased systolic blood pressure, pulse pressure, and central pulse pressure, among the hemodynamic parameters, indicate potential variations in vascular wall compliance and hemodynamic responses in the cardiovascular system. The increase in QT dispersion suggests that athletes may exhibit a heterogeneous repolarization process and an elevated risk of ventricular arrhythmias compared to the general population.

## 1. Introduction

The term “athlete’s heart” describes the structural and functional adaptations in the heart resulting from regular, intensive exercise [1]. These adaptations are commonly observed in athletes and are considered a normal physiological response to the increased demands of exercise on the cardiovascular system [2]. In recent years, “athlete’s heart” has been viewed as the heart’s natural adaptation, especially following the long-term effects of endurance exercises [3]. However, this concept also encompasses certain benefits, risks, and undesirable conditions associated with endurance sports and high-intensity training from past to present [1]. The athlete’s heart is characterized by changes in cardiac morphology, such as increased left ventricular size and wall thickness [2,4,5]. These changes vary depending on the type of exercise performed [2]. Endurance training, which involves prolonged periods of high cardiac output, is associated with increased diastolic load, leading to a larger cardiac chamber size. In contrast, short-duration “power” training, which is linked to a higher afterload and arterial blood pressure, results in an increased left ventricular wall thickness through compensatory concentric myocardial remodeling [6].

While most cardiac adaptations in athletes are deemed normal and beneficial, a small risk of underlying heart disease exists. Electrocardiogram (ECG) abnormalities in athletes may sometimes indicate hidden heart conditions, potentially increasing the risk of sudden cardiac death [7]. Therefore, distinguishing between physiological adaptations and pathological findings in athletes’ ECGs is essential [8]. Although the risk of heart attacks in athletes is generally lower compared to the general population, it is not entirely absent. Factors such as underlying cardiovascular conditions, genetic predisposition, and intense exercise regimens can elevate this risk [9].

Pulse wave velocity (PWV), a measure of arterial stiffness, is often used as an indicator of cardiovascular health [10]. It reflects the speed at which the pressure wave travels through the arteries, with higher values indicating a greater arterial stiffness [11]. Several studies have explored the relationship between the PWV and various cardiovascular parameters in athletes. For instance, one study found a significant negative correlation between the PWV and the maximal oxygen uptake (VO2max), suggesting that lower arterial stiffness is linked to better aerobic capacity [12,13]. This implies that arterial stiffness may influence cardiovascular adaptation and athletic performance [14].

In addition to the ongoing debate regarding whether the adaptations in the heart as a response to training are physiological and benign or pathological, potentially signaling disease or disability [15], it is also well known that the primary mechanism of sudden cardiac death in young competitive athletes is ventricular fibrillation [16].

QT dispersion, on the other hand, is a measure of the variability in the QT interval duration on an ECG [17]. An increased QT dispersion is thought to be associated with a higher risk of arrhythmias and sudden cardiac death [18]. However, in athletes, an increased QT dispersion compared to sedentary individuals is often observed, raising concerns about its potential as a marker for underlying heart conditions. A study focused on QT dispersion in athletes suggested that this increase is likely a physiological phenomenon related to the autonomic modulation of cardiac repolarization rather than a pathological marker [19]. This indicates that the increased QT dispersion in athletes is a normal adaptation to exercise training and does not necessarily signify an elevated risk of arrhythmias or cardiac events [11].

Despite numerous studies examining the cardiovascular health of athletes, there remains a gap in research concerning the simultaneous evaluation of the PWV and the QT dispersion across different athletic disciplines. Addressing this gap, the present study aims to explore the relationship between PWV and QT dispersion in various sports groups, providing a novel perspective on the physiological adaptations and potential risks associated with the athlete’s heart.

## 2. Materials and Methods

The endurance sports group consisted of 15 football players and 15 volleyball players, while the resistance sports group included 10 wrestlers and 10 judokas. All athletes had been training regularly for at least the past three years, with a minimum training frequency of four days per week and at least 10 h of training per week. Additionally, all participants were athletes who had competed in national and international competitions. This classification was made based on the predominance of aerobic metabolism in endurance sports and anaerobic energy systems in resistance sports, as supported by previous research [20,21]. Endurance training typically emphasizes aerobic capacity, which is crucial for sustained performance, whereas high-intensity resistance training focuses on strength development and uses anaerobic energy pathways to support short, intense bursts of effort. These different physiological demands are expected to influence cardiovascular parameters such as pulse wave velocity and QT dispersion, which are critical markers of vascular and cardiac health. The athletes were selected from among the students of the Faculty of Sports Sciences who met the inclusion criteria. Additionally, 30 sedentary men, aged 18–30 years and without any cardiac diseases, were included as the control group. The sample size was calculated using G*Power software (Germany), version 3.1.9.7. A medium effect size of 1.90, a significance level of 0.05, and a statistical power of 0.95 were applied, indicating that 50 experimental and 30 control subjects would be sufficient for this study. Athletes who participated in sports other than football, volleyball, wrestling, or judo, those under 18 or over 30 years of age, and individuals with cardiovascular or other diseases were excluded from this study.

All participants were informed about the experimental procedure and the purpose of this study, and their written informed consent was obtained. This study was approved by the 2020/696 ethics committee decision of the Ondokuz Mayıs University Clinical Research Ethics Committee. Moreover, this study was conducted in accordance with the ethical guidelines for human research in the Declaration of Helsinki, 2013.

Measurements of age, height, and body weight were recorded.

A 12-lead electrocardiogram (ECG) was performed on all subjects. For each lead, three QT intervals were measured, and their averages were calculated. QT dispersion was determined by calculating the difference between the longest and shortest average QT intervals obtained.

Arterial stiffness and cardiovascular hemodynamic parameters were measured using the Mobil-O-Graph 24 h ABPM NG^®^ (I.E.M. GmbH, Stolberg, Germany) arteriograph. This device was used to measure systolic blood pressure (SBP), diastolic blood pressure (DBP), central pulse pressure (cPP), mean arterial pressure (MAP), pulse pressure (PP), systolic z-score (zSys), diastolic z-score (zDia), augmentation index (AIx), total vascular resistance (TVR), PWV, cardiac output (CO), and stroke volume (SV). Measurements were taken from subjects at rest in a seated position using a cuff of appropriate size selected for each individual. The device’s cuff was automatically inflated to at least 35 mmHg above the measured brachial artery pressure to occlude the brachial artery. This occlusion halted blood flow for a maximum of 20 s during the measurement process. A membrane formed over the brachial artery beneath the upper edge of the inflated cuff, and pressure waves caused by central blood pressure changes were detected by sensitive pressure sensors embedded in the cuff. These signals were amplified and transmitted to the device’s specialized tonometer. The recorded waveforms were then analyzed using the HMS Client Server 5.1^®^ software, which was specifically designed for this device.

All measurements were conducted in the morning, at a time when athletes were fully rested.

### Statistical Analysis

Data were analyzed using the SPSS 27.0 software (IBM SPSS for Windows version 27, IBM Corporation, Armonk, NY, USA). Quantitative data were expressed as mean ± standard deviation. To determine whether the differences between the two groups (endurance and strength) were statistically significant, the independent samples *t* test was used as the parametric analysis method, assuming normality. In cases where the data did not meet the normality assumption, the Mann Whitney Test or Median Test, a non-parametric test, was applied to evaluate the differences between the same groups. To determine whether the differences between the three groups (control, endurance, and strength) were statistically significant, Fisher’s F Test (One Way ANOVA) was used as the parametric analysis method, assuming normality and homogeneity of variance. In cases where the data did not meet the normality assumption, the Kruskal–Wallis Test, a non-parametric test, was applied to evaluate the differences between the same groups. To assess the homogeneity of variances between the groups, the Levene Test was used before applying Fisher’s T Test and the Kruskal–Wallis Test. In cases where the variances between the groups were not homogeneous, the Tamhane Test was applied for multiple comparisons, while the Tukey Test was used when the variances were homogeneous, to identify which groups differed. The effect size of changes within each group was determined following the approach described by Kazis et al. (1989) [22]. The effect size was calculated using the formula “(EB) = difference between measurements/standard deviation of the first measurement”. Effect sizes were categorized as “small” for values between 0.20 and 0.50, “moderate” for values between 0.51 and 0.80, and “large” for values of 0.81 and above [23]. The statistical significance level was set at *p* < 0.05. A 95% confidence level was adopted for all analyses, and a *p*-value of less than 0.05 was considered statistically significant.

## 3. Results

According to Table 1, with 95% confidence, there were no statistically significant differences between the groups in terms of age and height, while significant differences were found in weight and body mass index (BMI) between the control group, endurance group, and resistance group. The results of the multiple comparison test revealed that the difference in weight was due to the resistance group–control group (*p* = 0.001) and the endurance group–control group (*p* = 0.006). Similarly, multiple comparison analyses were performed to determine which groups contributed to the differences in BMI. However, since Fisher’s F test is a parametric analysis method, it is necessary to check both the normality assumption and the homogeneity of variances between groups. The Levene test indicated that the variances between the groups were not homogeneous. Based on this result, the Tamhane test was selected for the multiple comparison analysis. According to the Tamhane test, the differences in BMI were found to be due to the resistance group–control group (*p* = 0.013) and the endurance group–control group (*p* = 0.001).

According to Table 2, with 95% confidence, there were no statistically significant differences between the groups in terms of the QT interval and QTc values. In contrast, significant differences were found in the QT dispersion between the control, endurance, and resistance groups. The results of the multiple comparison test indicated that this difference in the QT dispersion was due to the control group–endurance group (*p* = 0.018) and the control group–resistance group (*p* = 0.012) with 95% confidence. According to the effect size analyses, the effect size was found to be small (d < 0.2) for the QT interval across all groups, while it was moderate (0.2 ≤ d < 0.5) for the QT dispersion in all groups. In contrast, the corrected QTc interval showed a small effect only in the control group, whereas a moderate effect was observed in the other two groups.

No significant differences were found between the athletes and the control group in terms of the arterial parameters. Additionally, according to the effect size analyses, the effect size for the pulse wave velocity and the AIx variables was found to be small (d < 0.2) across all groups (Table 3).

Significant increases in the SBP, cPP, and PP values were found in the athlete groups. The results of this test indicated that the differences in the SBP between the groups were due to the control–endurance (*p* = 0.025) and the control–resistance (*p* = 0.025) groups. Additionally, multiple comparison analyses were performed to determine which groups contributed to the differences in the PP and cPP values. However, since Fisher’s F test is a parametric analysis method, it is necessary to check both the normality assumption and the homogeneity of the variances between the groups. This was assessed using the Levene test, which showed that the variances between the groups were homogeneous with 95% confidence. Based on this result, the Dunnett test was selected for the multiple comparison analysis [24]. According to the Dunnett test, the differences in both the PP and cPP were found to be due to the resistance–control (*p* = 0.036 and *p* = 0.019) groups. Additionally, according to the effect size analyses, the effect size for the DBP was small (d < 0.2) across all groups, while it was moderate (0.2 ≤ d < 0.5) for the SBP in all groups. For the MAP, a moderate effect was observed only in the control group, whereas the effect size was small in the other groups. For the PP and cPP variables, a small effect was observed in the endurance group, while the effect size was moderate in the other groups. Regarding the zSystolic variable, a small effect was found in the resistance group, whereas the effect size was moderate in the other groups. For the zDiastolic, SV, and total vascular TVR variables, a moderate effect was observed in the resistance group, while the effect size was small in the other two groups (Table 4).

The results of the multiple comparison tests conducted for the groups with significant differences are presented in Table 5. According to the results of the multiple comparison tests shown in Table 5, the differences in the QT dispersion variable were found to be due to the control group–endurance group (*p* = 0.018) and the control group–resistance group (*p* = 0.012) at a 95% confidence level. The effect size between the control and endurance groups (d = −2.68) indicated a very large effect. A similar effect was observed between the control and resistance groups. However, the effect size between the endurance and resistance groups (d = −0.43) indicated a small-to-moderate effect, supporting the possibility of no significant difference between these two groups, meaning that they were similar. In this case, the differences were attributed to the control group having lower values compared to both the endurance and resistance groups.

Similarly, the differences in the SBP variable between the groups were found to be due to the control group–endurance group (*p* = 0.025) and the control group–resistance group (*p* = 0.025). When examining the effect sizes, it can also be stated that, similar to the QT dispersion, the differences in the SBP were due to the control group having lower values compared to both the endurance and resistance groups.

Furthermore, multiple comparison analyses were conducted to determine which groups contributed to the differences in the PP and cPP variables. However, since Fisher’s F test is a parametric analysis method, it was necessary to examine both the normality assumption and the homogeneity of the variances between the groups. This examination was performed using the Levene test, which indicated that the group variances were homogeneous at a 95% confidence level. Based on this result, the Tukey test was chosen for the multiple comparison analysis. According to the Tukey test, the differences in both the PP and cPP variables were found to be due to the resistance group–control group comparisons (p_PP = 0.036; p_cPP = 0.019, *p* < 0.05). Examining the effect sizes for the pairwise comparisons of both variables, it was observed that the differences in the PP and cPP were also due to the control group having lower values compared to both the endurance and resistance groups.

## 4. Discussion

The incidence of sudden cardiac death (SCD) among athletes has been reported to be 1:63,682 athlete-years, with a higher incidence observed in male athletes compared to female athletes [25]. In athletes engaged in intense training, structural adaptations of the heart, often referred to as the “athlete’s heart”, lead to significant changes that can differ from the typical heart structure observed in non-athletes. These changes, which may exceed normal physiological limits, can create diagnostic challenges during echocardiographic assessments [4].

Left ventricular hypertrophy (LVH) is a physiological adaptation frequently observed in athletes due to prolonged physical activity. However, differentiating physiological LVH from pathological hypertrophy is crucial, particularly in conditions such as hypertrophic cardiomyopathy (HCM), which is a leading cause of sudden cardiac death (SCD) in athletes. This differentiation often involves a combination of diagnostic tools, including echocardiography (ECHO), patient history, and advanced imaging techniques. Such a comprehensive approach is vital to identifying pathological LVH and preventing adverse outcomes [26].

Cardiac hypertrophy, particularly in athletes, can be a physiological adaptation to exercise, but pathological hypertrophy, such as hypertrophic cardiomyopathy (HCM), can lead to sudden cardiac death. When left ventricular wall thickness reaches 13–15 mm, distinguishing between physiological hypertrophy and HCM can be challenging. Imaging techniques such as echocardiography play a crucial role in making this distinction. Additionally, exercise improves cardiac function, enhances vascular health, and reduces age-related arterial stiffness. While exercise is a key tool in managing cardiovascular diseases, overtraining and doping can have adverse effects, especially in athletes [27,28,29].

In athletes, approximately 95% of sudden deaths are heart-related, with hypertrophic cardiomyopathy being the leading cause [30,31]. Cardiovascular-related deaths are reported to be six times higher in high-intensity exercise compared to low-intensity exercise. In a long-term follow-up study, it was found that individuals running 6–12 miles per week at a speed of 6 km/h had a 38% risk of death, while those running at speeds of 8 km/h or higher and covering 20 miles per week had a sixfold higher risk of death [32]. Looking at cardiac biomarkers, endurance exercises have been shown to cause damage to the heart. Serum troponin and B-type natriuretic peptide (BNP) levels increase by 50% in circulation. Increased oxidative stress at the tissue level contributes to this damage. Left ventricular damage is observed in continuous running for six hours, and as the duration and intensity of the run increase, the damage becomes more pronounced. These changes are also observed in the right ventricle and can persist even one week after the run [33].

Functional hypertrophy that develops in response to training is distinct from pathological hypertrophy caused by chronic diseases. In physiological hypertrophy, no impairment in left ventricular function is observed. While the hearts of elite athletes are larger than those of sedentary individuals, their size generally remains within the upper limits of normal when adjusted for body size or increased end-diastolic volume. To date, no compelling scientific evidence has shown that a specific exercise training regimen harms the normal heart. On the contrary, it has been demonstrated that the cardiac functional capacity, stroke volume, and peak cardiac output of athletes are significantly higher than those of healthy sedentary individuals [30,34]. However, there are insufficient data to determine whether physiological hypertrophy could be a cause of sudden death [35,36]. On the other hand, changes occurring in the athlete’s heart cannot be entirely accepted as a physiological process, as the possibility of negative outcomes cannot be ruled out [4].

Various ECG parameters are being studied for this purpose, as they reflect the electrical activity of the heart. The QT interval on an ECG encompasses the time from ventricular activation to the electrical recovery following activation. The QT duration can change when the heart rate increases or decreases. Therefore, in order to eliminate the effect of the heart rate, the QT interval is calculated as “corrected”, and this corrected value is referred to as the QTc. A QTc value of less than 450 ms for men and less than 460 ms for women is generally considered normal [37]. QT dispersion is a measurement used to non-invasively show the disruption of homogeneity in myocardial repolarization. It is calculated by measuring the difference between the longest and shortest QT intervals on a 12-lead ECG [38]. It is considered a marker of myocardial electrical instability and a predictor of arrhythmic events [39] In normal individuals, the QT dispersion ranges between 20 and 50 ms [38]. In heart failure, the QT dispersion is significantly increased [40].

Arterial stiffness reflects the rigidity and flexibility of the vessel walls. To assess arterial stiffness, the PWV, AIx, and central blood pressure (CBP) are used [41,42].

Pulse wave analysis, including parameters such as the PWV and AIx, is a useful tool for the non-invasive assessment of central hemodynamics and arterial elasticity indices by analyzing the arterial pressure waveforms [43,44]. The PWV measures the speed of the pressure waves traveling along arterial segments, serving as a parameter that reflects the arterial stiffness over a specific distance [45]. However, oscillometric methods, such as the Mobil-O-Graph device, assess this parameter in terms of the peripheral arterial stiffness, in contrast to traditional carotid–femoral PWV measurement techniques. The device analyzes both the forward and reflected components of pressure waves to compute the data. AIx refers to the change in the pulse pressure magnitude caused by the reflected wave and is considered an important marker of hemodynamic conditions related to arterial stiffness. The Mobil-O-Graph device, unlike traditional methods, evaluates peripheral arterial stiffness while providing indirect information about central arterial stiffness. Although the device offers valuable data on arterial stiffness and hemodynamic parameters, it is important to note that the results obtained cannot be directly compared to those from conventional methods. Therefore, a careful consideration of the measurement methodology and the limitations of the Mobil-O-Graph is crucial when interpreting the findings [46].

Our study offers important insights into arterial stiffness and hemodynamic parameters in athletes. Nevertheless, no significant differences were found between the athlete groups in terms of arterial stiffness and hemodynamic parameters. The literature suggests that there may be cardiovascular differences between endurance and strength athletes. For instance, a study by Tomschi et al. (2021) found that endurance athletes had lower systolic blood pressure and arterial stiffness values compared to strength and team sport athletes [47]. The lack of significant differences in our study could be attributed to factors such as sample size, study design, or the training history of the athletes. Future research exploring these differences in more detail could help further elucidate the impact of training type on arterial health.

In this study, the QT dispersion, QT interval, QTc, arterial stiffness, and hemodynamic cardiac parameters were investigated in male athletes who engaged in regular exercise compared to a sedentary control group.

In our study, the lower body mass index (BMI) observed in the athletes reflects the positive effects of regular training on body composition. Particularly in strength-based sports, a higher lean body mass is commonly observed among athletes, which may have a positive impact on metabolic health and contribute to a reduction in cardiovascular risk factors [48]. It is a common approach to use body fat percentage as a more accurate measure of body composition. However, in this study, only BMI data were available, and body fat percentage data were not included in our analysis. Nevertheless, the finding that the athletes had lower BMI values compared to the control group highlights the role of consistent training in shaping body composition and is consistent with the existing literature.

Furthermore, our study provides valuable insights into arterial stiffness (PWV and AIx) and hemodynamic parameters in male athletes. It is important to note that potential sex differences in these parameters should be considered. Prior research indicates that the effects of aerobic and anaerobic exercise on blood pressure and arterial stiffness may differ between males and females, likely due to hormonal influences and structural differences in vascular physiology. For instance, a study by Collier (2008) found sex-specific responses in arterial stiffness following different exercise modalities [49]. Additionally, more recent research has explored sex differences in arterial stiffness and hemodynamic responses to exercise, emphasizing the need for further investigation in this area [50]. Therefore, future studies that include female athletes are essential to fully elucidate the impact of sex on arterial stiffness and hemodynamic responses.

An increase in the QT dispersion in athletes has been associated with the structural and electrical changes in the heart that occur with regular training. Recent studies have indicated that the QT dispersion may be higher in endurance athletes, which could suggest a condition similar to the physiological adaptations observed in systemic hypertension [51,52]. Sports such as football, volleyball, wrestling, and judo can create different stress factors on the heart, potentially increasing this dispersion. Recent studies have demonstrated that the prolongation of the QT interval and dispersion detected through surface ECGs are used as markers of ventricular repolarization abnormalities in various cardiac conditions [53,54]. Recent studies have shown that an increase in the QT dispersion in veteran athletes without cardiovascular disease is associated with an increase in left ventricular mass, suggesting that athletic cardiac hypertrophy in veteran athletes may partially result from residual hypertrophy, rather than being entirely physiological [55,56]. These findings suggest that regular training can lead to myocardial adaptations, resulting in greater heterogeneity in the repolarization processes. An increase in the QT dispersion is considered an indicator of an electrical imbalance in the heart, which may elevate the risk of arrhythmias [54]. However, other evidence suggests that this increase observed in athletes is a physiological adaptation rather than a pathological condition [57]. Studies propose that an increase in the QT dispersion reflects non-homogeneous myocardial repolarization, which could elevate the likelihood of arrhythmias. Nevertheless, whether this increase in the QT dispersion leads to long-term cardiac risks remains controversial. Endurance and strength-based sports are known to induce structural and functional changes in the athlete’s heart. This phenomenon, commonly referred to as “athlete’s heart”, may involve electrical changes such as left ventricular hypertrophy, increased vagal tone, and bradycardia [4].

No significant differences were observed between the athletes and the control group in terms of the PWV, DBP, MAP, zSys, zDia, CO, SV, TVR, and AIx. This indicates that, although the athletes’ cardiovascular systems may have adapted in certain aspects, they exhibited similar characteristics to the control group regarding arterial stiffness and vascular elasticity. While the PWV is widely recognized as a marker of cardiovascular health, it did not demonstrate a significant difference compared to the QT dispersion. The lack of significant differences in the pulse wave velocity and other cardiac parameters suggests that cardiovascular responses in athletes may vary individually, reflecting the complex interplay of training adaptations and inherent physiological characteristics [58,59]. The effects of exercise on arterial stiffness remain a controversial topic. However, the systolic blood pressure, pulse pressure (PP), and central pulse pressure (cPP) were significantly higher in the athletes compared to the control group. Increases in these parameters may reflect hemodynamic adaptations resulting from the athletes’ intense physical training, highlighting the cardiovascular system’s response to sustained physiological demands [60]. These changes, particularly observed in endurance training, increase the heart’s capacity to pump blood at higher pressures, indicating the vascular system’s adaptation to this condition. A rise in the pulse pressure may lead to higher pressure fluctuations in the vascular system, a phenomenon that may be more pronounced in endurance athletes. A high central pulse pressure (cPP) could indicate that the heart expends more force when pumping blood from the center to the peripheral vessels. Recent studies have suggested that prolonged endurance exercise can lead to permanent changes in arterial stiffness, potentially contributing to an increase in pulse pressure [47]. The exposure to high pressure during exercise suggests that it is not arterial stiffness alone but also hemodynamic pressure responses that adapt. These findings highlight that, despite no significant difference in arterial stiffness, the cardiovascular system may adapt to specific pressure changes. As a result, the elevated systolic blood pressure and pulse pressure observed in athletes should be considered important parameters in cardiovascular risk assessments [33,61].

The findings of this study make significant contributions to understanding the effects of exercise on cardiac electrical stability. The observed increase in the QT dispersion in the athletes can be considered an indicator of electrical adaptations in the heart and heterogeneity in cardiac repolarization processes. Furthermore, the lack of a significant difference in the PWV results, which assess arterial stiffness, between the athletes and the control group suggests that certain aspects of the cardiovascular systems in athletes may share similar characteristics with sedentary individuals. This indicates that, rather than arterial stiffness, hemodynamic pressure responses may show a more pronounced adaptation with exercise. However, large-scale, prospective studies are needed to better understand whether the increase in the QT dispersion in athletes leads to long-term cardiac risks and the long-term effects of thePWV on cardiovascular health. Additionally, advanced electrophysiological evaluations are recommended to clarify the relationship between the QT dispersion and cardiac arrhythmias.

## 5. Conclusions

This study evaluated the differences in the QT dispersion, QT interval, QTc, and cardiovascular hemodynamic parameters between endurance- and resistance-trained athletes and control groups. Our findings show statistically significant differences in the QT dispersion, systolic blood pressure, pulse pressure, and central pulse pressure (cPP). This suggests that the type of exercise may influence both the cardiovascular and electrophysiological variables. In contrast, the lack of significant differences in the pulse wave velocity between the groups indicates that some vascular characteristics may remain constant across different types of training.

The results obtained suggest that the differences observed between the athlete groups may be attributed to sport-specific physiological adaptations. In light of these findings, it is recommended that the QT dispersion be considered, particularly in cardiovascular risk assessments.

The cross-sectional design of this study and the fact that it only included male participants limit the generalizability of the findings. Future studies involving different groups and long-term designs could provide further insights into this area. This study highlights the importance of targeted protective and monitoring strategies for athletes in sports where endurance and resistance training are applied at varying levels.

## Figures and Tables

**Table 1:** Comparisons of demographic information of individuals.

Variable	Groups			*p*
	Control X ± SD	Endurance X ± SD	Strength X ± SD	
**Age (yr)**	21.07 ± 1.799	20.17 ± 1.913	20.75 ± 1.954	0.050
**Height (m)**	1.77 ± 0.041	1.75 ± 0.066	1.79 ± 0.086	0.097
**Weight (kg)**	78.43 ± 8.716	68.87 ± 6.601	72.25 ± 13.514	**0.001**
**BMI (kg/m^2^)**	24.83 ± 2.229	22.47 ± 2.319	22.26 ± 3.273	**0.001**

BMI: body mass index; X: mean; SD: standard deviation; *p* < 0.05.

**Table 2:** Measurement results of ECG of the groups.

Variable	X ± SD	*p*	Effect Size (d)
**QT dispersion (msn)**		0.016	
**Control**	40.67 ± 5.208		−0.425
**Endurance**	44.67 ± 6.288		0.205
**Strength**	45.5 ± 6.863		0.335
**QTc (msn)**		0.279	
**Control**	391.13 ± 16.334		0.123
**Endurance**	383.80 ± 17.393		−0.268
**Strength**	392.90 ± 22.893		0.218
**QT (msn)**		0.404	
**Control**	368.10 ± 17.165		0.165
**Endurance**	360.80 ± 24.616		−0.183
**Strength**	365.20 ± 20.255		0.027

QTc: corrected QT interval; QT: QT interval; X: mean; SD: standard deviation; *p* < 0.05.

**Table 3:** The arterial stiffness parameter measurement results for the groups.

Variable	X ± SD	*p*	Effect Size (d)
**PWV (m/s)**		0.458	
**Control**	4.98 ± 0.302		−0.181
**Endurance**	5.08 ± 0.381		0.120
**Strength**	5.06 ± 0.298		0.060
**Alx (%)**		0.861	
**Control**	14.13 ± 7.895		0.061
**Endurance**	13.70 ± 7.809		0.005
**Strength**	12.90 ± 7.650		−0.098

PWV: pulse wave velocity; Alx: augmentation index; X: mean; SD: standard deviation; *p* < 0.05.

**Table 4:** The cardiovascular hemodynamic parameter measurement results for the groups.

Variable	X ± SD	*p*	Effect Size (d)
**SBP (** **mmHg)**	121.19 ± 11.158	0.031	
**Control**	117.20 ± 9.799		**−** **0.358**
**Endurance**	123.53 ± 11.834		**0.210**
**Strength**	123.65 ± 10.825		**0.220**
**DBP (** **mmHg)**	71.15 ± 9.220	0.590	
**Control**	71.30 ± 9.337		0.016
**Endurance**	71.97 ± 9.122		0.089
**Strength**	69.70 ± 9.493		−0.157
**MAP (** **mmHg)**	94 ± 8.251	0.308	
**Control**	92.23 ± 7.546		−0.215
**Endurance**	95.47 ± 8.788		0.178
**Strength**	94.45 ± 8.351		0.05
**PP (** **mmHg)**	50.27 ± 11.761	**0.039**	
**Control**	46.10 ± 11.562		**−0.355**
**Endurance**	51.97 ± 11.346		**0.145**
**Strength**	54.00 ± 11.314		**0.317**
**zSys (** **mmHg)**	119.45 ± 9.968	0.076	
**Control**	116.43 ± 10.241		−0.303
**Endurance**	122.23 ± 9.435		0.279
**Strength**	119.80 ± 9.512		0.035
**zDia (** **mmHg)**	73.12 ± 8.585	0.213	
**Control**	73.70 ± 6.899		0.068
**Endurance**	74.47 ± 8.593		0.157
**Strength**	70.25 ± 10.442		−0.334
**cPP (** **mmHg)**	46.89 ± 10.962	**0.021**	
**Control**	42.67 ± 11.211		**−0.385**
**Endurance**	48.57 ± 8.705		**0.153**
**Strength**	50.70 ± 12.014		**0.348**
**CO (L/min)**	5.49 ± 0.821	0.361	
**Control**	5.37 ± 0.769		−0.146
**Endurance**	5.47 ± 0.753		−0.024
**Strength**	5.71 ± 0.980		0.268
**SV (mL)**	72.73 ± 15.545	0.327	
**Control**	70.16 ± 14.597		−0.165
**Endurance**	72.54 ± 14.557		−0.012
**Strength**	76.84 ± 18.095		0.264
**TVR (s × mg/mL)**	1.05 ± 0.147	0.433	
**Control**	1.05 ± 0.161		0.000
**Endurance**	1.06 ± 0.122		0.068
**Strength**	1.01 ± 0.159		−0.272

SBP: systolic blood pressure; DBP: diastolic blood pressure; MAP: mean arterial pressure; PP: pulse pressure; zSys: systolic z-score; zDia: diastolic z-score; cPP: central pulse pressure; CO: cardiac output; SV: stroke volume; TVR: total vascular resistance; X: mean; SD: standard deviation; *p* < 0.05.

**Table 5:** Multiple comparison results for the control, endurance, and resistance groups.

Variable	*Sd_pooled_*	*p*	Effect Size (d)
**QT dispersion (msn)**			
**Endurance group–Strength group**	1.917	0.692	-0.43
**Endurance group–Control group**	1.491	0.018	−2.68
**Strength group–Control group**	1.805	0.012	−2.68
**SBP (** **mmHg)**			
**Endurance group–Strength group**	3.304	0.814	0.00
**Endurance group–Control group**	2.805	0.025	−2.26
**Strength group–Control group**	2.950	0.025	−2.19
**PP (** **mmHg)**			
**Endurance group–Strength group**	3.272	0.811	−0.62
**Endurance group–Control group**	2.958	0.122	−1.98
**Strength group–Control group**	3.310	0.049	−2.39
**cPP**			
**Endurance group–Strength group**	2.929	0.764	−0.73
**Endurance group–Control group**	2.591	0.084	−2.28
**Strength group–Control group**	3.330	0.027	−2.41

SBP: systolic blood pressure; PP: pulse pressure; cPP: central pulse pressure; Sd: standard deviation; *p* < 0.05.

## Data Availability

Data is contained within the article.
